# Genomic alterations in oral multiple primary cancers

**DOI:** 10.1038/s41368-023-00265-w

**Published:** 2024-02-18

**Authors:** Xuan Zhou, Xinjia Cai, Fengyang Jing, Xuefen Li, Jianyun Zhang, Heyu Zhang, Tiejun Li

**Affiliations:** 1 Department of Oral Pathology, Peking University School and Hospital of Stomatology & National Center of Stomatology & National Clinical Research Center for Oral Diseases & National Engineering Laboratory for Digital and Material Technology of Stomatology & Beijing Key Laboratory of Digital Stomatology & Research Center of Engineering and Technology for Computerized Dentistry Ministry of Health & NMPA Key Laboratory for Dental Materials, Beijing, China; 2 Research Unit of Precision Pathologic Diagnosis in Tumors of the Oral and Maxillofacial Regions, Chinese Academy of Medical Sciences (2019RU034), Beijing, China; 3 Central Laboratory, Peking University School and Hospital of Stomatology, Beijing, China

**Keywords:** Oral cancer detection, Cancer genetics

## Abstract

Oral squamous cell carcinoma (OSCC) is the predominant type of oral cancer, while some patients may develop oral multiple primary cancers (MPCs) with unclear etiology. This study aimed to investigate the clinicopathological characteristics and genomic alterations of oral MPCs. Clinicopathological data from patients with oral single primary carcinoma (SPC, *n* = 202) and oral MPCs (*n* = 34) were collected and compared. Copy number alteration (CNA) analysis was conducted to identify chromosomal-instability differences among oral MPCs, recurrent OSCC cases, and OSCC patients with lymph node metastasis. Whole-exome sequencing was employed to identify potential unique gene mutations in oral MPCs patients. Additionally, CNA and phylogenetic tree analyses were used to gain preliminary insights into the molecular characteristics of different primary tumors within individual patients. Our findings revealed that, in contrast to oral SPC, females predominated the oral MPCs (70.59%), while smoking and alcohol use were not frequent in MPCs. Moreover, long-term survival outcomes were poorer in oral MPCs. From a CNA perspective, no significant differences were observed between oral MPCs patients and those with recurrence and lymph node metastasis. In addition to commonly mutated genes such as *CASP8*, *TP53* and *MUC16*, in oral MPCs we also detected relatively rare mutations, such as *HS3ST6* and *RFPL4A*. Furthermore, this study also demonstrated that most MPCs patients exhibited similarities in certain genomic regions within individuals, and distinct differences of the similarity degree were observed between synchronous and metachronous oral MPCs.

## Introduction

Oral squamous cell carcinoma (OSCC) is the most prevalent histological subtype of oral cancer, encompassing malignancies that occur in the buccal mucosa, tongue, gingiva, larynx, palate, or lip. The clinical manifestations of OSCC vary depending on the specific cancer type, with some patients presenting with nonhealing nodules or sores in the oral mucosa.^1^ Others with tumors located in the pharynx or floor of the mouth may experience difficulties in swallowing or changes in vocal quality.^2^ Approximately 75% of OSCC are attributed to alcohol and tobacco use.^3^ Extensive research has been conducted on OSCC, including studies investigating its clinical presentations, etiology, and treatment strategies.

However, there exist a subset of patients who develop multiple primary cancers (MPCs) in the oral cavity, and the causes of this phenomenon remain unclear. The incidence of MPCs in OSCC ranges from 10 to 34% and contributes significantly to cancer-related mortality.^4^ In 1932, Warren and Gates proposed diagnostic criteria for MPCs.^5^ Currently, the most commonly utilized definitions are provided by the Surveillance Epidemiology and End Results (SEER) project and the International Association of Cancer Registries and the International Agency for Research on Cancer (IACR/IARC).^6,7^ The difference in diagnostic criteria between these two systems primarily lies in the statistical analysis of tumor locations. For example, while SEER treats tumors occurring in different parts of the colon as separate entities, the colon is considered a single location in the IACR/IARC system. Additionally, SEER recommends a 2-month interval to differentiate synchronous and metachronous MPCs, whereas the IACR/IARC suggests a cutoff of 6 months to classify tumors as synchronous or metachronous based on the time interval. Numerous investigations have illuminated disparities in the prognosis of synchronous and metachronous cancers. Shiga et al., in a Japanese study, unveiled that synchronous OSCC presents a notably inferior 5-year survival rate compared to its metachronous counterpart.^8^ This observation concurs with the findings of a 2019 study by Bugter et al., wherein the 5-year survival rates for synchronous and metachronous cancers stood at 25% and 85%, respectively.^9^ Scholars hold varying opinions regarding the prognosis of single primary cancer (SPC) and MPCs. Some scholars discovered that as the number of tumor occurrences increased, the prognosis for patients worsened, while others found that there was no difference in prognosis between metachronous MPCs and SPC.^10,11^

With the advancement of modern sequencing technologies, Braakhuis et al. proposed the utilization of molecular diagnostic techniques to accurately identify “True” MPCs in 2002.^12^ It was postulated that “True” MPCs should exhibit distinct molecular characteristics; however, assessing the degree of similarity between two tumors remains a challenging task. Particularly in cases of malignant tumors with the same histological type occurring in the same organ, distinguishing between primary cancer and metastatic cancer has always posed a dilemma. The IASLC Lung Cancer Staging Project suggests that it is easier to identify differences between the two tumors rather than similarities.^13^ The presence of shared characteristics does not definitively indicate that they are the same. Few features can be considered definitive, and many commonly utilized criteria are suggestive but prone to misclassification.

To elucidate the development of oral MPCs, Slaughter et al. introduced the concept of “cancerization of field”. These events encompass multi-step and complex processes involving genetic alterations, damage induced by carcinogens such as tobacco and alcohol, human papillomavirus infection, and other unidentified factors. Researchers have endeavored to identify unique molecular features of secondary oral primary cancers to aid in clinical diagnosis, such as tumor suppressor genes (*p53, p14, p73*), *FAS/FASLG*, *p21*, *p27*, and oncogenes (*MDM2*, *MDM4*), which may serve as effective molecular markers for MPCs.^14,15,16,17,18^ Furthermore, aberrant methylation levels of *CCNA1* and *TIMP3* have also been implicated in the development of MPCs.^19^ However, conclusive evidence supporting these associations is currently lacking. Previously, it was believed that tumors frequently exhibit copy number alteration (CNA) events. However, recent reports indicated that the presence of chromosomal aneuploidies in different chromosomes within tumors may potentially serve as a prognostic indicator for distinct tumor outcomes, and could potentially inhibit tumor development.^20^

In this study, we established a stringent definition of oral MPCs and conducted a comprehensive clinicopathological analysis. Based on this definition, we employed low-depth CNA sequencing and whole-exome sequencing (WES) approaches to explore the molecular distinctions between synchronous and metachronous cancers in different MPCs patients.

## Result

### Clinicopathological differences between MPCs and SPCs

**Fig. 1:**
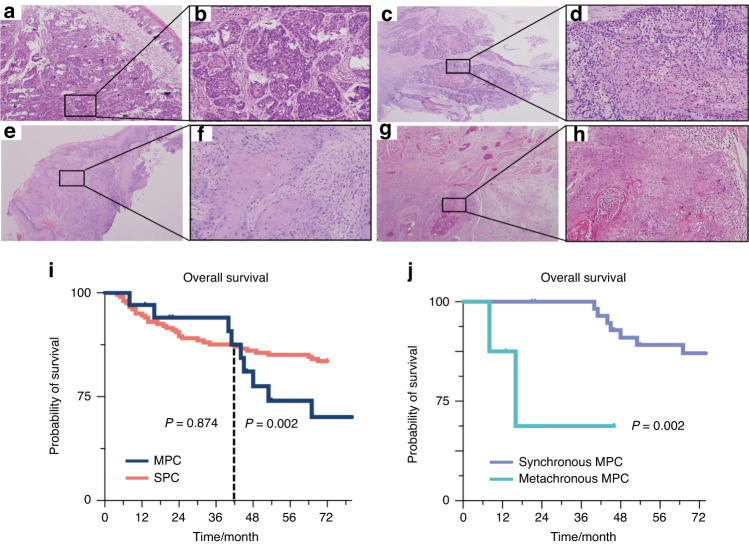
Representative microphotographs of oral MPCs lesions and survival curves for oral MPCs patients. **a**–**d** These four images illustrate microphotographs of synchronous MPCs P34. **a**, **b** reveal palate SCC (T1), while **c**, **d** display tongue SCC (T2). **e**–**h** present HE slices of patients with metachronous MPCs P02. **e**, **f** exhibit tongue SCC (T1), and **g**, **h** reveal buccal SCC (T2). Original magnification: ×40 (**a**, **c**, **e**, **g**); ×200 (**b**, **d**, **f**, **h**). **i** Kaplan–Meier survival analysis of 34 MPCs patients and 202 SPC patients. **j** Kaplan–Meier survival analysis of 4 patients with synchronous MPCs and 30 patients with metachronous MPCs

A total of 34 MPCs patients were included in our study. The clinical profiles of all 34 patients were summarized in Supplementary Table 1. Among these patients, 4 were synchronous MPCs, while 30 were metachronous MPCs including three patients with tumors in three distinct locations. Representative microphotographs of synchronous and metachronous MPCs were shown in Fig. 1a–h. Patient P34 presented with synchronous cancers, manifesting tumors in the palate and tongue, discovered in close succession within a 3-month period at the age of 59. In contrast, Patient P02 was diagnosed with metachronous cancers. His initial diagnosis occurred at age 36 when he developed tongue SCC. After 42 months, a second diagnosis revealed buccal SCC. The mean interval between the initial diagnosis of OSCC and the subsequent tumor in metachronous MPCs was 56 months, ranging from 10 to 144 months.

After a 6-year follow-up, we collected data from 202 SPC patients to investigate the differences between MPCs and SPC patients. The clinicopathological parameters were analyzed and compared (Table 1). While there was no significant difference in the age at the time of initial diagnosis, a notable gender disparity was observed between the SPC and MPCs groups (*P* < 0.001). The majority of SPC patients were male (130/202; 64.36%), whereas 70.59% (24/34) of MPCs patients were female. Additionally, we examined the smoking and drinking habits of the two patient groups and found that the proportion of smokers and drinkers was significantly lower in the MPCs group compared to the SPC group.

**Table 1:** Comparison of clinicopathological information between MPC and SPC

Items	MPCs	SPC	*P*
Patients	34	202	
Gender			
Male	10 (29.41%)	130 (64.36%)	<0.001
Female	24 (70.59%)	72 (35.64%)	
The mean age at 1st diagnosis (year)	60	58	=0.418
Smoking			
Yes	6 (17.65%)	122 (60.40%)	<0.001
No	28 (82.35%)	80 (39.60%)	
Drinking			
Yes	4 (11.76%)	61 (30.20%)	<0.001
No	30 (88.24%)	141 (69.80%)	
Tumors	71	202	
Tumor size			
T1	66 (92.96%)	93 (46.04%)	<0.001
T2 + T3 + T4	5 (7.04%)	109 (53.96%)	
Lymph node metastases			
Yes	13 (18.31%)	78 (38.61%)	=0.002
No	58 (81.69%)	124 (61.39%)	
Pathology grade			
Well	66 (92.96%)	123 (60.89%)	<0.001
Poor	5 (7.04%)	79 (39.11%)	
Location			
Gingiva	25 (35.21%)	40 (19.80%)	<0.001
Buccal mucosa	15 (21.13%)	22 (10.89%)	
Tongue	18 (25.35%)	79 (39.11%)	
Lip	5 (7.04%)	8 (3.96%)	
Palate	5 (7.04%)	7 (3.47%)	
Others	3 (4.23%)	46 (22.77%)	

*MPC* multiple primary cancers, *SPC* single primary cancer, *Others* oropharynx, floor of mouth, jawbone, retromolar

There were 71 tumors from 34 MPCs patients. Regarding tumor location, tongue was the most common location in the SPC group, accounting for 39.11% (79/202) of cases, while gingiva was the predominant location in MPCs patients (25/71; 35.21%). There was a significant difference in tumor size between the SPC and MPCs groups. In the MPCs group, most tumors were categorized as T1 stage, with only four tumors falling into the T2 stage category. In contrast, within the SPC group, cases spanned from T1 to T4 (*P* < 0.001). The incidence of lymph node metastasis was significantly lower in the MPCs group compared to the SPC group (*P* < 0.001). During our follow-up period, we found that one synchronous cancer patient (P30) had distant metastasis, while two SPC patients had distant metastasis. Furthermore, MPCs patients had a better initial tumor pathology grade compared to SPC patients (*P* < 0.001).

Although there was no significant difference in the age at the time of initial diagnosis during the early stage (within 42 months), the overall survival rate for MPCs initially appeared to be better than that for SPC (*P* = 0.874), but it subsequently became worse than SPC after 42 months (*P* = 0.002, Fig. 1i). Although the sample size of synchronous MPCs patients was restricted, the prognosis of synchronous MPCs patients was significantly worse than that of metachronous MPCs patients (*P* = 0.002) based on the Kaplan–Meier curve (Fig. 1j).

### Copy number alteration profiles of different tumors in the same patient

**Fig. 2:**
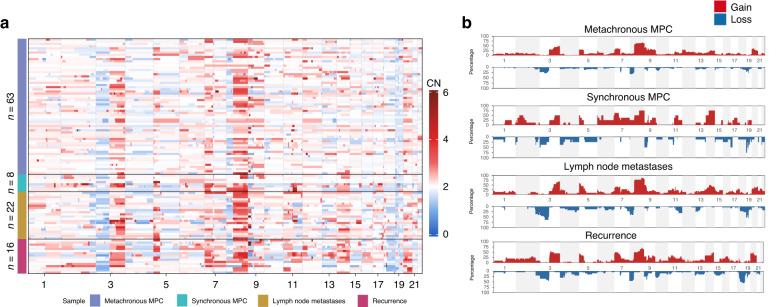
Comparison of CNA among MPCs, lymph node metastatic and recurrent OSCC cohorts. **a** CNA profiles of 71 samples from 34 MPCs patients (including 63 samples from metachronous MPCs and 8 samples from synchronous MPCs), 22 from 11 patients with lymph node metastases, and 16 from 8 recurrent patients. CN copy number. **b** Cumulative copy number frequencies for MPCs, lymph node metastatic and recurrent OSCC cohorts. The *y*-axis displays the percentage of samples harboring CNAs in the four groups. Red indicates CNA gain events, while blue represents CNA loss events

We collected a total of 71 tumor samples from 34 MPCs patients, 16 tumor samples from 8 recurrent patients, and 22 tumor samples from 11 lymph node metastasis patients for CNA sequencing profiles (Supplementary Table 1). The heatmap and frequency map revealed the presence of CNA events in all chromosomes (Fig. 2a, b). Notably, Chr7, Chr8, Chr9, Chr11, and Chr14 exhibited a high frequency of copy number gain events, while copy number losses were predominantly observed at a low frequency on Chr4 and Chr18. Additionally, Chr3 and Chr8 displayed breakpoints in all four groups. The hotspot regions described in MPCs patients were consistent with those previously reported in head and neck squamous cell carcinoma (HNSCC), lung SCC, and esophageal SCC and were also observed in recurrence and lymph node metastasis patients in our study.^21,22,23^

**Fig. 3:**
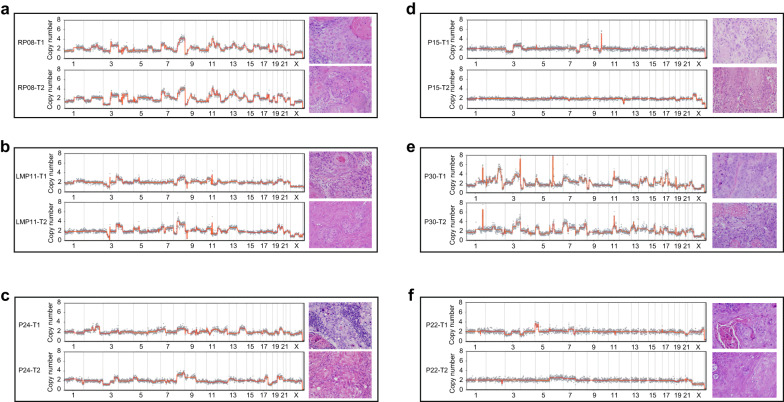
CNA plots for representative MPCs, lymph node metastatic and recurrent OSCC samples. **a** CNA plots for two tumors of RP08 patient. “RP” denotes recurrent patients. **b** CNA plots for two tumors of LMP11 patient. “LMP” signifies lymph node metastasis patients. **c**, **d** CNA plots of tumors from two metachronous MPCs patients. **e**, **f** CNA plots of tumors from two synchronous MPCs patients. HE images were captured at ×400 magnification

Subsequently, we analyzed the CNA patterns of different tumors within the same patient. We found that for all recurrent tumor patients, both the primary and recurrent cancers exhibited a consistent CNA change of over 75% (range: 75%–100%) in same patient. For instance, CNA patterns of the initial tongue tumor (RP08-T1) and the recurrent tumor (RP08-T2) that appeared 8 months later in the recurrent patient RP08 exhibited a high degree of similarity, with an 78% match. They shared common regions on Chr 1, 2, 3, 4, 6, 7, 8, 9, 11, 12, 13, 14, 15, 16, 18, 19, 20, and 21, with subtle distinctions observed on Chr 5 and 10 (Fig. 3a). Similarly, in all patients with lymph node metastasis, both primary cancer and the metastatic lymph node cancer exhibited a consistent CNA pattern of over 70% (range: 70–92%) in the same individuals. For example, the lymph node metastasis patient LMP11 exhibited common regions of CNA in Chr3, Chr5, Chr8, Chr10, Chr11, Chr20, Chr21, and Chr22 in both the primary tumor (LMP11-T1) and the lymph node tumor (LMP11-T2) (Fig. 3b). These indicate a high degree of similarity in CNA pattern profiles between primary and recurrent cancer, as well as between primary cancer and metastatic cancer.

In metachronous MPCs, we observed two scenarios in the CNA patterns. In 66.67% (20/30) of patients, tumors in different locations within the same patient displayed similar CNA regions. For instance, patient P24 was diagnosed with tumors in the left upper gingiva (P24-T1) and right buccal mucosa (P24-T2) with an interval of 88 months (Fig. 3c). The CNA results revealed common hotspots in Chr3, Chr8, and Chr20, as well as other distinct CNA events. Specifically, P24-T1 exhibited amplification in Chr2, Chr11, and Chr14, while the amplification region in P24-T2 was found in Chr5p. On the other hand, in 33.33% (10/30) of MPCs patients, the CNA profiles differed significantly between tumors in different locations within the same patient. For example, patient P15 presented with tumors in the buccal mucosa (P15-T1) and lip (P15-T2), with a 14-month interval between the two. P15-T1 exhibited CNA events in Chr3, Chr8, and Chr10, whereas the other tumor displayed different alterations in Chr12 (Fig. 3d). The degree of CNA similarity did not exhibit a consistent trend based on the temporal or spatial distance.

Similarly, in synchronous MPCs, two situations were observed. Three out of four patients demonstrated higher similarity in their CNA patterns. For instance, patient P30 had tumors in the lower gingiva (P30-T1) and pharynx (P30-T2) simultaneously, and the two tumors shared CNA events on Chr1, Chr3, Chr5, Chr6, Chr7, Chr8, Chr11, Chr15, Chr17, Chr19, and Chr22 (Fig. 3e). However, patient P22, who had tongue SCC (P22-T1) and gingival SCC (P22-T2) as synchronous MPCs, exhibited mostly distinct CNA patterns (Fig. 3f). CNA events were observed in Chr3, Chr4, Chr5, Chr7 and Chr21 in P22-T1 but not in P22-T2.

### Whole-exome sequencing in MPCs patients

WES was performed on individual tumors and normal tissues from four MPCs patients (P02, P12, P20, P34). The average number of reads per tumor was 67,870,702, with a ratio of high-quality clean reads ranging from 96.57 to 98.57%. Germline variants were removed by comparing the tumor data to the normal tissue data. Across all collected tumors, an average of 131,976 single nucleotide polymorphisms (SNPs) and 16,613 insertions and deletions (Indels) were observed per tumor (Supplementary Table 2).

**Fig. 4:**
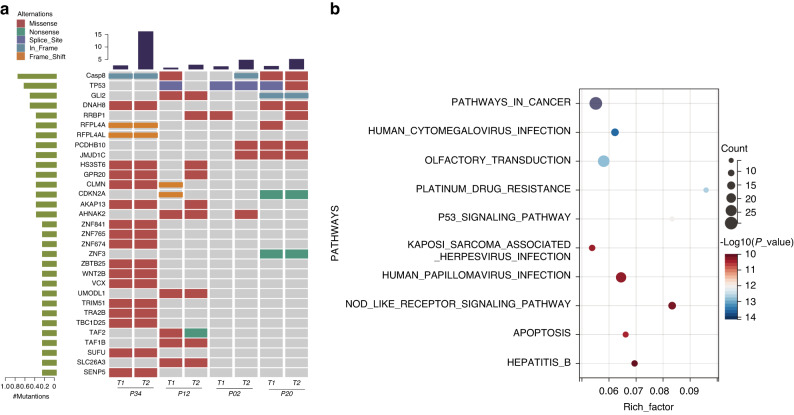
The WES data results for four MPCs patients. **a** Top 30 SMGs in MPCs patients. The middle panel displays somatic mutations by patient (column) and by gene (row) with different colors representing distinct mutation types. The top histogram depicts the number of accumulated mutations in each individual sample. **b** The significant KEGG top 10 pathways in MPCs patients

The most common type of transition mutation in the tumors was C > T, observed in all samples (Supplementary Fig. 1). Regarding the tumor mutational burden (TMB), the mean TMB value for all lesions was 2.0 mutations per megabyte (MB), ranging from 0.94 to 3.94 mutations per MB (Supplementary Table 3). We focused on significantly mutated genes (SMGs) using MuSiC and identified a total of 96 SMGs (Supplementary Table 4). *CASP8*, an apoptosis-related gene, exhibited a mutation frequency of 75.00% (6/8), including three missense SNV (c.G700A:p.G234R, c.C773A:p.T258N, c.C773A:p.T258N) and one stopgain site mutations (c.C1348T:p.Q450X) in three tumors (P12-T1, P20-T1 and P20-T2). The remaining samples (P02-T2, P34-T1 and P34-T2) showed non-frameshift deletions in amino acids 277–279 of the *CASP8* gene. All *CASP8* gene mutations were located in the same protein domain (Peptidase_C14) (Supplementary Fig. 2a). The top 2–4 genes with mutations were *TP53* (63.00%), *DNAH8* (50.00%), and *GLI2* (50.00%) (Fig. 4a and Supplementary Table 4). Additionally, mutations in AHNAK2, *GPR20, PCDHB10*, *CDKN2A*, *JMJD1C*, *AKAP13*, and *RRBP1* were also detected with a frequency of 37.50% (Supplementary Table 4). Although the sample size in our study was relatively small, we identified genes with high-frequency mutations that differ from those previously reported in OSCC, suggesting the presence of unexplored genetic changes in MPCs patients that may contribute to disease development.^24^ To assess the feasibility of targeted drug interventions for the identified gene mutations, our analysis delved into the SMG dataset employing genes cataloged within the Therapeutic Target Database (TTD). The results unveiled the presence of 20 distinct genes that harbor potential as therapeutic targets, meticulously detailed in Supplementary Table 5. Furthermore, 47 genes directly associated with the immune system were found in all four patients (Supplementary Table 6).

In addition to known OSCC driver genes (*TP53* and *CDKN2A*), our analysis revealed several genes not previously implicated in cancer.^24^ The *HS3ST6* mutation was found in three samples from two patients, all occurring in the Sulfotransfer_1 structural domain (Supplementary Fig. 2b). The *RFPL4A* mutation was observed in two patients (three samples) in the SPRY domain, with a Polyphen score of 0.973, indicating harmful mutations (Supplementary Fig. 2c).

Pathway analysis using Kyoto Encyclopedia of Genes and Genomes (KEGG) with the mutated genes in MPCs revealed the top three pathways as Pathways in cancer, Human cytomegalovirus infection, and Olfactory transduction (Fig. 4b and Supplementary Table 7).

### Phylogenetic tree analysis in MPCs patients

**Fig. 5:**
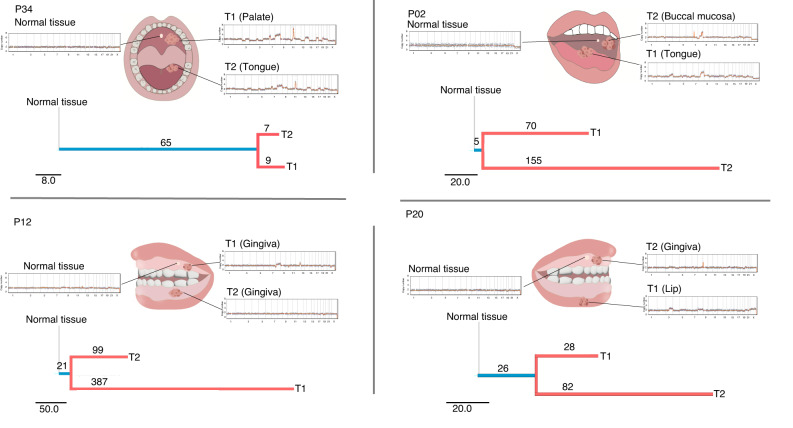
Four MPCs patients, each having different disease locations and corresponding phylogenetic trees, were depicted along with displays of copy number alteration plots. *T* tumor

To gain insights into the genetic phylogeny of MPCs, we conducted a phylogenetic tree analysis based on SNVs and compared their CNA profiles (Fig. 5 and Supplementary Table 8). The branch length in the phylogenetic trees represented the number of mutations in the corresponding tumors, and the trunk mutations referred to the mutations shared by two tumors. Despite variations in the number of trunk mutations, ranging from 5 to 65, all four patients exhibited clear evidence of shared gene mutations.

Patient P34, who had synchronous MPCs, had the longest trunk length (*n* = 65). Furthermore, the CNA profiles of the two tumors in this patient showed a high degree of similarity, specifically in Chr3, Chr 5, Chr 7, Chr 8, Chr 11, Chr 14, Chr 17, and Chr 19. These findings indicate a close relationship between the two tumors in synchronous MPCs (Fig. 5).

In patients with metachronous MPCs, the length of trunk mutations was shorter compared to branch mutations (Supplementary Fig. 3). When considering both the CNA profiles and phylogenetic tree analysis of these three patients, the overall similarity was not significant. Trunk mutations ranged from 3 to 48%. Notably, we observed more unique changes between the two tumors in each individual.

## Discussion

In our study, we identified distinct clinical and pathological differences between MPCs and traditional OSCC. Interestingly, we observed a higher incidence of MPCs in females, while smoking and alcohol were not identified as risk factors. Furthermore, the affected sites in MPCs were predominantly the buccal mucosa and gingiva, which differed from the most common site in traditional oral cancer, which is the tongue.^25^ These findings suggest that the risk factors for oral MPCs may differ from those of traditional oral cancer. OSCC is more prevalent among men, a trend that aligns with the gender distribution observed in our analysis of SPC patients. Notably, we observed a higher incidence of oral MPCs among women. Gender ratios have been reported differently in the research. Remarkably, our study revealed a distinct gender ratio when compared to a prior study on oral MPCs conducted in Hong Kong, despite consistent patterns in the common sites of occurrence.^26^ Moreover, Jovanov et al. carried out a subsequent investigation involving 727 patients suffering from OSCC. Their findings revealed that individuals with primary SCC in the lip and oral cavity exhibit a notably heightened susceptibility to developing oral and/or pharyngeal cancer compared to the general population. Interestingly, it appears that women within the oral cancer demographic may face an even greater risk of MPCs.^27^ Moreover, some researchers have identified an even higher proportion (55.6%) of women among patients with MPCs, highlighting a robust association between HNSCC and esophageal cancer.^28^ However, these disparities may also be attributed to variations in patient cohorts, diverse methods of collecting data on MPCs, or even mere chance distinctions between these studies. Some research indicated that MPCs may be associated with proliferative verrucous leukoplakia (PVL), while PVL often occurs in women.^29^^,30^ The development of MPCs has been suggested to be associated with genetic susceptibility, and further research is needed to explore its relationship with gender.^31^

The prognosis of MPCs compared to SPC remains controversial. Li et al. reported a poorer 5-year survival rate for MPCs compared to SPC.^32^ In our study, we found that the prognosis of MPCs needs to be assessed separately. In the early stage (around less than 42 months), the prognosis of MPCs was slightly better than that of SPC, although not significantly. However, over time and with the impact of multiple cancer occurrences, the prognosis of MPCs became worse than that of SPC. This finding is consistent with the survival analysis conducted by Cai et al. based on the SEER database for MPCs.^33^ We also observed that the prognosis of synchronous MPCs was significantly worse than that of metachronous MPCs, which aligns with reports on lung MPCs.^34^ Additionally, synchronous and metachronous cancers may exhibit differences in their pathogenic mechanisms. In the context of colorectal MPCs, researchers suggest that synchronous cancers may be influenced by individual factors such as smoking and alcohol consumption.^35^ In contrast, metachronous cancers may be linked to familial genetic factors, as exemplified by conditions like Familial Adenomatous Polyposis.^36^ Metachronous cancers could potentially arise due to specific genetic predispositions and may manifest over an individual’s lifetime. Conversely, synchronous cancers appear to result more from specific environmental factors causing damage within a particular temporal window.^37^ Furthermore, there are differences in the treatment of MPCs. Chang et al. found that in cases of lung MPCs with identical EGFR mutations, tyrosine kinase inhibitors for EGFR-targeted therapy may provide a possibility for inoperable patients with lung MPCs. This presents a new approach for the management of various other primary cancers.^38^ The existence of different tumor microenvironments within the same patient may lead to complexities in treatment.^39^

We did not observe statistically significant differences in CNA events between different groups, including patients with recurrence, lymph node metastasis, and MPCs. Twenty years ago, some scholars proposed that the absence of 3p14 and 9p21 can serve as a simple diagnostic tool for oral MPCs.^40^ However, SCC, including HNSCC, lung SCC, and esophageal SCC, share common hotspot regions such as Chr 1, Chr 3, Chr 5, Chr 8, Chr 9, and Chr 11, which align with our CNA results (including MPCs, recurrence and lymph node metastasis patients).^21^^,22^^,23^ MPCs may not have distinctive CNA pattern that can be used to distinct it from SPC. The reasons for the diverse occurrence of aneuploidies CNA in tumors of MPC patients remain unclear, and the functional implications of CNA events in tumor patients are still to be explored.^20^

Furthermore, in our attempt to identify potential pathogenic genes for oral MPCs, we found that in addition to commonly detected gene mutations such as *TP53, CASP8*, and *MUC16*, there were also relatively rare gene mutations compared to OSCC or HNSCC. The SMGs identified in our study did not entirely match the results reported by Li et al. for oral MPCs, except for *TP53* and *MUC16*, which were also identified as highly frequent mutation genes.^32^ This discrepancy may be due to the small sample size in both studies, including only 9 patients with a second primary cancer in that research and 8 samples from 4 patients in our study. Many of these rare mutation genes have unknown functions in OSCC, although some have sporadically appeared in other cancer contexts. Identifying these genes in OSCC may also open doors to potential targeted therapies. The *RFPL4A* gene, previously linked to colorectal cancer, has gained attention in Japanese research for its potential role in chemotherapy resistance in G1 phase individuals.^41^ This discovery suggests *RFPL4A* could be a new target for challenging cancer diseases. *DNAH8*, found to be upregulated in metastatic prostate cancer tissue, is associated with higher tumor recurrence and metastasis risk in patients with elevated expression, potentially serving as a novel regulator of the androgen receptor (AR) linked to metastatic tumors and adverse prognoses.^42^ *AHNAK2*, overexpressed in various cancer types like renal, pancreatic, melanoma, and lung adenocarcinoma, correlates with poor outcomes.^43^ Studies in papillary thyroid carcinoma suggest *AHNAK2* might act as a tumor suppressor gene.^44^ Elevated *AHNAK2* expression relates to advanced clinical staging, increased risk of lymph node metastasis, and poorer overall survival. Research by Kim et al. hints at *AHNAK2*’s role in the immune microenvironment, indicating its downstream relationship with *CXCL16*, associated with immune cell infiltration and adverse outcomes in thyroid carcinoma.^45^ In lung adenocarcinoma, *AHNAK2* is considered an independent prognostic indicator.^46^ Furthermore, *RRBP1* emerges as a potential oncogene, closely associated with poor prognosis in lung adenocarcinoma.^47^ The *E2F1/RRBP1* pathway may drive proliferation and metastasis in hepatocellular carcinoma.^48^ In therapeutic studies, elevated *RRBP1* expression is seen in chemotherapy-resistant OSCC tumor samples compared to chemotherapy-responsive tumors and inhibiting *RRBP1* expression may restore cisplatin-induced cell death in chemotherapy-resistant OSCC patients, significantly reducing tumor burden.^49^

The study of molecular clonality among different tumors in MPCs has been an ongoing area of research. Wang et al. analyzed the clonality of different lesions in lung MPCs using three methods: loss of heterozygosity, *TP53* mutation screening analyses, and X-chromosome inactivation data.^50^ They found that 77% of lung MPCs were clonally related. Similarly, clonality was also observed in liver MPCs.^51^ In a recent case report by Ba et al., common-driven genes were used to distinguish between lung MPCs and satellite nodules with lung metastasis.^52^ They concluded that different tumors in the same patient had distinct driver mutations, suggesting different molecular events driving the two tumors and influencing subsequent treatment approaches. Molecular methods have also been employed to differentiate recurrent cancer from MPCs in oral cancer. A study conducted in Taiwan utilized WES to sequence 15 patients with oral MPCs and identified driver genes and trunk mutations as distinguishing factors. The number of trunk mutations varied among MPCs cases, while recurrent patients exhibited completely duplicated mutated genes.^1^ However, the study did not differentiate between synchronous and metachronous MPCs, nor did it consider prognosis.

We did not observe a shared mutation gene in the trunk mutations among the four patients during the analysis of the phylogenetic tree. When comparing different tumors within the same patients we found that some metachronous cancer patients displayed distinct CNA patterns, while others exhibited varying degrees of CNA similarity. The similarity between synchronous MPCs was higher, and this pattern was more evident in patients with recurrence and lymph node metastasis, which aligns with the results reported by Scholes et al.^53^ Additionally, both the CNA similarity analysis and phylogenetic tree analysis demonstrated consistent similarity among synchronous cancers. However, the results of CNA similarity analysis and phylogenetic tree analysis did not always align. For example, while CNA results categorized P02 as belonging to “some similar types”, the phylogenetic tree analysis showed the lowest number of common gene mutations and no driven genes as common genes. There were also cases where the CNA results were completely inconsistent, but shared gene mutations were present (e.g., P12). It is possible that both CNA and trunk gene mutation methods can be used to evaluate the similarity of MPCs. On the other hand, this result also suggested the difficulty in identifying common driver genes in patients with oral MPCs, as patients exhibit significant individual variability. We have observed that various patients tend to possess distinct trunk genes, which could signify the critical involvement of this gene in the pathogenesis of individual, thus potentially emerging as prospective targets for personalized molecular therapies. Therefore, our conclusions based on CNA and WES suggest that synchronous cancers exhibit more similar genetic changes at the molecular level, potentially leaning toward treatment with the same targeted drugs, while metachronous cancers show greater differences and may lean toward more complex target treatment approaches. Further investigation and attention are required to explore this disease comprehensively.

Gaining a deeper and more comprehensive comprehension of oral MPCs not only aids in distinguishing between patients with recurrence and metastasis from those with MPCs, but also opens up treatment possibilities for the latter group, ultimately reducing unnecessary utilization of medical resources. Presently, research on oral MPCs is constrained, encompassing both clinical pathology and molecular investigations, thereby posing challenges in employing a singular diagnostic tool for accurate detection. Comparable to other MPCs (e.g., lung), diverse molecular diagnostic criteria have been proposed, yet the consensus among experts remains rooted in the notion that distinguishing between a single primary and a metastatic tumor cannot solely rely on a single quantitative method.^13^ The situation is akin to oral MPCs, where a comprehensive examination of various perspectives is required to elucidate the distinguishing characteristics of a singular primary tumor or MPCs.

This study has some limitations, including a small sample size for synchronous cancer and being a single-center study, which may have introduced bias into the final results. At the same time, due to the small sample size, it is not possible to effectively integrate molecular research and clinical information. The underlying causes of oral MPCs are still unclear, as is the genetic relationship between the two tumors. Relying solely on individual molecular methods to diagnose the presence of MPCs is likely not feasible, and all evidence should be considered indicative. A comprehensive judgment based on clinical and pathological manifestations, as well as chromosomal changes and mutation genes, may be necessary.

## Methods and material

### Patient cohort and materials

This study was conducted in accordance with the Declaration of Helsinki and was approved by the Ethics Committee of Peking University Hospital of Stomatology (PKUSSIRB-201949116). A retrospective search for patients with MPCs was conducted at Peking University Hospital of Stomatology from 2000 to 2021. The focus of this study was on multiple primary OSCCs. We selected patients with OSCC that occurred in different locations, defined according to the criteria determined by Warren and Gates in 1932. The tumor locations were coded based on the anatomic location using the International Classification of Diseases, 10th Revision (ICD-10). The interval between MPCs can be divided into simultaneous cancer (interval ≤6 months) and metachronous cancer (interval >6 months) by IACR/IARC system.

Simultaneously, OSCC patients diagnosed with recurrence or lymph node metastasis were also included. The diagnostic criteria for recurrence followed the Odense Birmingham definition,^54^ and lymph node metastasis was determined based on lymph node dissection during primary cancer surgery. Tumor size and lymph node positivity were coded according to the latest UICC TNM classification.^55^ All patients included in the study had formalin-fixed and paraffin-embedded (FFPE) samples or frozen tissue available for analysis.

Additionally, clinical-pathological data of patients with SPC who visited Peking University Hospital of Stomatology in 2017 and had 6-year follow-up information were collected.

### Sample selection and copy number alteration sequencing

FFPE samples from different locations within the oral cavity (lymph node samples for metastasis patients) were collected for CNA sequencing, following previous reports.^56^ Briefly, a 10 μm section was prepared using REM710 (Yamato, Japan) and then subjected to UV laser cutting using LMD7 (Leica) to capture tissues, ensuring a cell count between 300–600 per sample. Tissues were lysed, and all libraries were sequenced using an Illumina HiSeq 4000 sequencer with PE150 sequencing strategy. The 2 × 150 paired-end reads were trimmed using Cutadapt (version 2.10) to remove adapters and aligned to the human reference genome (hg19) using the Bowtie2 aligner (version 2.2.9). Non-overlapping dynamic bins with 1 Mb resolution were generated across the genome. The bin counts were obtained, and CNA calling was performed using the circular binary segmentation (CBS) algorithm. We approximate the degree of similarity between the two tumors by assessing the occurrence of similar CNA events in a specific chromosomal segment. The sequencing depth for each sample was ~0.3 Gb (0.1×). The major parameters used to filter out low-quality samples were median absolute pairwise difference (MAPD) and the number of mapping reads (samples with MAPD < 0.3 and reads >100 000 were considered of qualified quality).

### Whole-exome sequencing and data analysis

Fresh tissue samples stored at −80 °C were used for DNA extraction using the QIAamp Blood and Tissue DNA Kit (QIAamp, Germany). WES was performed using the Agilent SureSelect Human All Exon V6 kit (Agilent, USA) to capture and enrich exome sequences. High-throughput sequencing was conducted on Illumina platforms with PE150 strategy at Novogene Bioinformatics Technology Co., Ltd (Beijing, China).

Total read numbers, raw data, error rates, and the percentage of reads with Q30 scores (the percent of bases with Phred-scaled quality scores greater than 30) were calculated and summarized. The filtered reads were mapped to the reference genome (b37) using Burrows-Wheeler Aligner (BWA) software and Samblaster to generate a BAM file. Germline single nucleotide polymorphisms (SNPs) and InDels were called using SAMtools with the following filter parameters: QUAL ≥ 20, DV ≥ 4, MQ ≥ 30. SNV mutations were detected using MuTect, and somatic InDels were identified by Strelka.^57,58,59,60,61^ Variant annotation was performed using ANNOVAR. MuSiC and KEGG were employed for selecting SMGs and pathway annotation, respectively.^62,63,64^ Phylogenetic trees were constructed using Phylip-3.695 to compare non-synonymous gene mutations. Polyphen-2, a tool for predicting the deleterious effects of gene mutations, was employed. A higher Polyphen-2 score indicates a greater level of harm (http://genetics.bwh.harvard.edu/pph2/index.shtml). Immune-related genes were obtained from the IMMUPORT database (https://www.immport.org/home). The target druggability characteristics were acquired from the Therapeutic Target Database (TTD) (https://db.idrblab.net/ttd/).^65^ Select variants were validated with Sanger sequencing (Supplementary Table 9).

### Statistical methods

Statistical analysis was conducted using IBM SPSS Statistics 25.0 (IBM Corp., Armonk, NY, USA) and R 4.1.1. Clinical data, including age, gender, locations of oral cancer, smoking history, and drinking history, were compared between MPCs and SPC groups using the Mann–Whitney *U* test, Chi-square test, as appropriate. The survival rate of MPCs and SPC patients was calculated using the Kaplan–Meier method, and we compared the survival curves using a log-rank test to test differences in survival probabilities across group. Statistical significance was defined as a *P* value <0.05.

### Supplementary information

Supplemental Figure 1

Supplemental Figure 2

Supplemental Figure 3

Supplemental Table 1

Supplemental Table 2

Supplemental Table 3

Supplemental Table 4

Supplemental Table 5

Supplemental Table 6

Supplemental Table 7

Supplemental Table 8

Supplemental Table 9
