# Supplementary figures and images for: Genomic alterations in oral multiple primary cancers
Source: Int J Oral Sci. 2024 Feb 18;16:13. doi: 10.1038/s41368-023-00265-w (PMC10874441; doi:10.1038/s41368-023-00265-w)

Fraction of Mutations

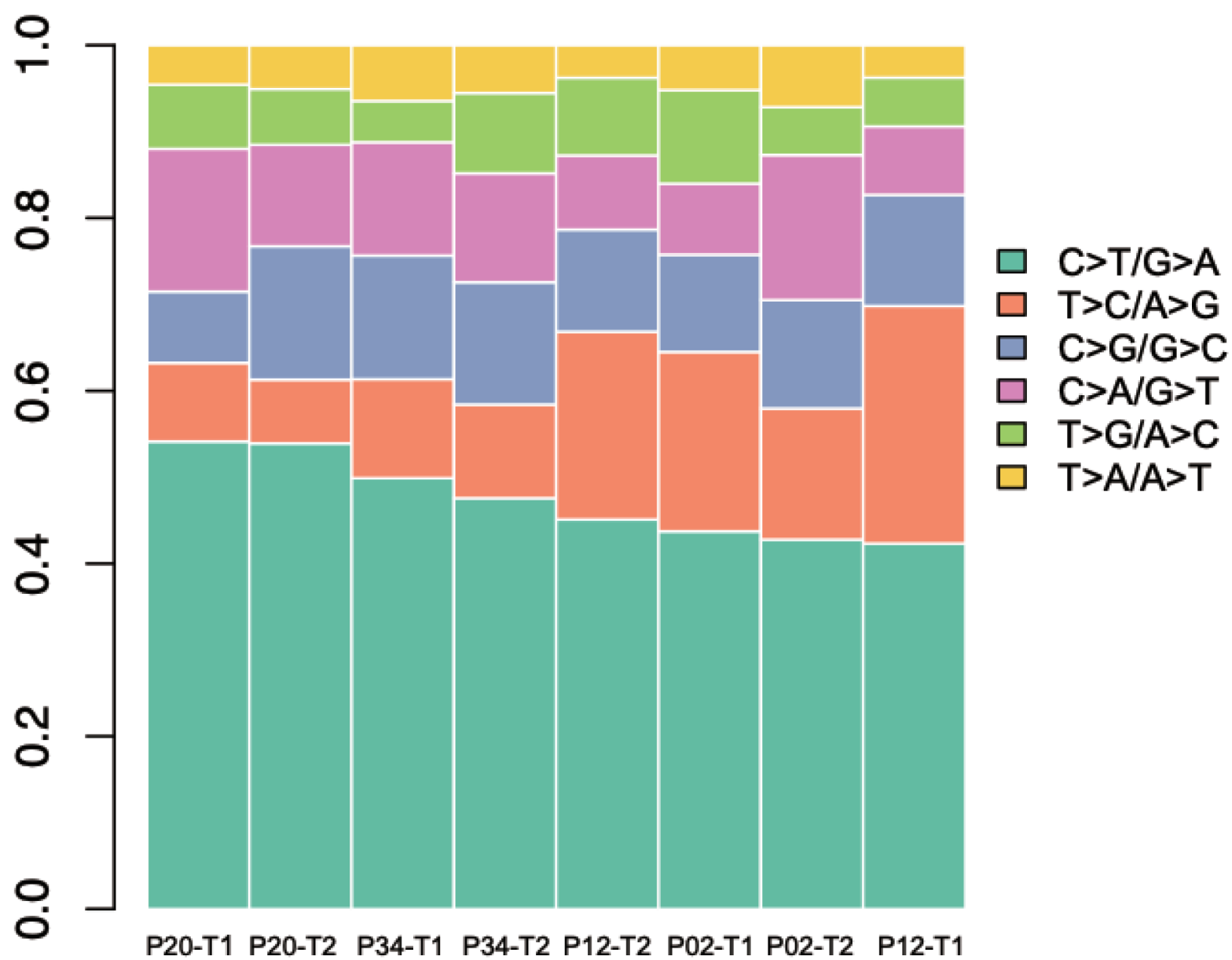

Supplement: Supplementary file 1 — Supplemental Figure 1 [file 41368_2023_265_MOESM1_ESM.pdf]

a

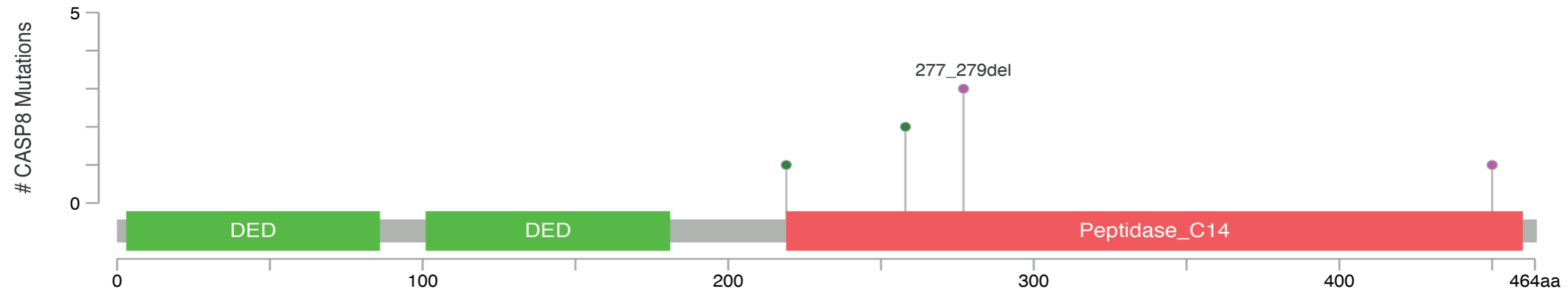

b

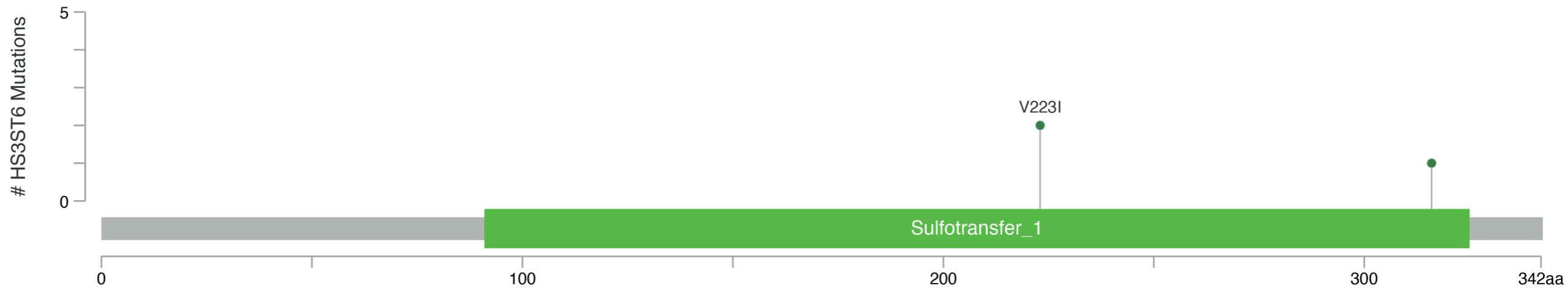

c

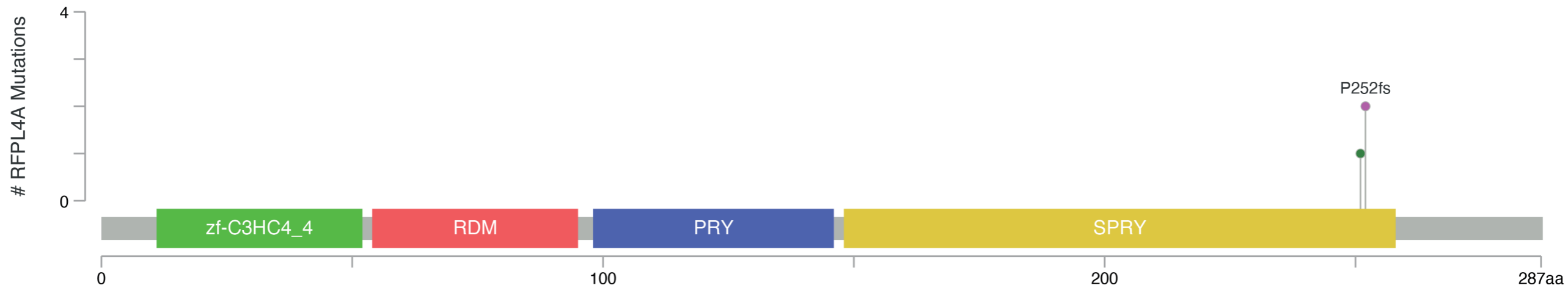

Supplement: Supplementary file 2 — Supplemental Figure 2 [file 41368_2023_265_MOESM2_ESM.pdf]

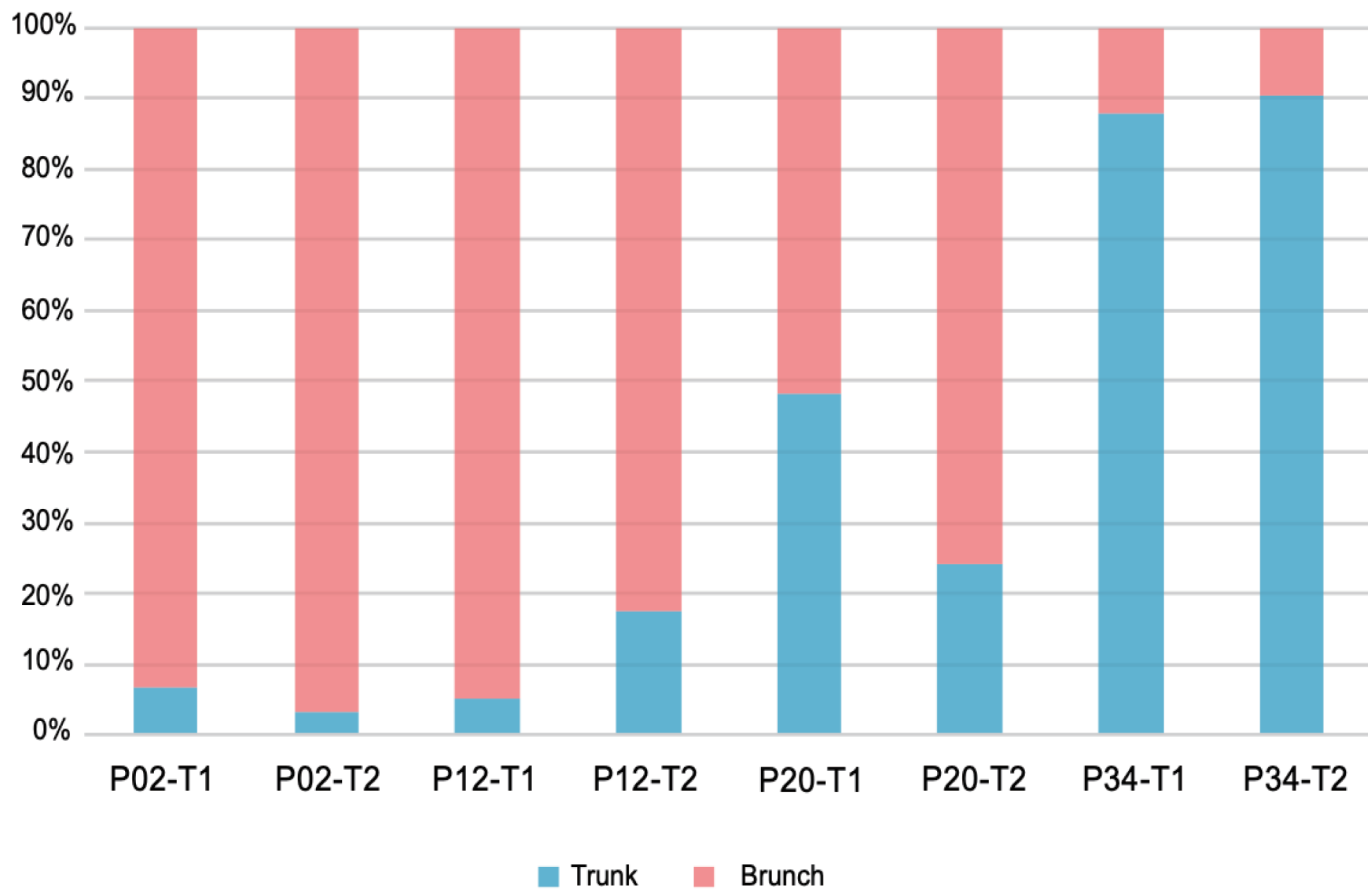

Supplement: Supplementary file 3 — Supplemental Figure 3 [file 41368_2023_265_MOESM3_ESM.pdf]
